# Acute Effects of Two Different Species of Amyloid-*β* on Oscillatory Activity and Synaptic Plasticity in the Commissural CA3-CA1 Circuit of the Hippocampus

**DOI:** 10.1155/2020/8869526

**Published:** 2020-12-18

**Authors:** Cécile Gauthier-Umaña, Jonathan Muñoz-Cabrera, Mario Valderrama, Alejandro Múnera, Mauricio O. Nava-Mesa

**Affiliations:** ^1^Neuroscience Research Group (NEUROS), Escuela de Medicina y Ciencias de la Salud, Universidad del Rosario, Bogotá, Colombia; ^2^Behavioral Neurophysiology Laboratory, Physiological Sciences Department, School of Medicine, Universidad Nacional de Colombia, Bogotá, Colombia; ^3^Deparment of Biomedical Engineering, Universidad de los Andes, Bogotá, Colombia

## Abstract

Recent evidence indicates that soluble amyloid-*β* (A*β*) species induce imbalances in excitatory and inhibitory transmission, resulting in neural network functional impairment and cognitive deficits during early stages of Alzheimer's disease (AD). To evaluate the *in vivo* effects of two soluble A*β* species (A*β*_25-35_ and A*β*_1-40_) on commissural CA3-to-CA1 (cCA3-to-CA1) synaptic transmission and plasticity, and CA1 oscillatory activity, we used acute intrahippocampal microinjections in adult anaesthetized male Wistar rats. Soluble A*β* microinjection increased cCA3-to-CA1 synaptic variability without significant changes in synaptic efficiency. High-frequency CA3 stimulation was rendered inefficient by soluble A*β* intrahippocampal injection to induce long-term potentiation and to enhance synaptic variability in CA1, contrasting with what was observed in vehicle-injected subjects. Although soluble A*β* microinjection significantly increased the relative power of *γ*-band and ripple oscillations and significantly shifted the average vector of *θ*-to-*γ* phase-amplitude coupling (PAC) in CA1, it prevented *θ*-to-*γ* PAC shift induced by high-frequency CA3 stimulation, opposite to what was observed in vehicle-injected animals. These results provide further evidence that soluble A*β* species induce synaptic dysfunction causing abnormal synaptic variability, impaired long-term plasticity, and deviant oscillatory activity, leading to network activity derailment in the hippocampus.

## 1. Introduction

Alzheimer's disease (AD), the most common type of dementia and progressive neurodegenerative disorder worldwide, is characterized by selective neuronal loss, and two histopathological features in *postmortem* tissue are extracellular amyloid plaques composed of amyloid beta peptide (A*β*) and intracellular neurofibrillary tangles composed of hyperphosphorylated tau protein [1]. Recent evidence indicates that soluble forms of A*β* induce glutamatergic, cholinergic, and GABAergic imbalance, resulting in functional impairment of neural networks during early AD stages [2–5]. In fact, A*β*-induced synaptic dysfunction precedes selective neuronal degeneration and may explain memory impairment during early AD stages and mild cognitive impairment, a prodromal stage of AD [6, 7]. Although therapies based on modulation of GABAergic neurotransmission have been proposed for AD [8], current symptomatic therapies include cholinesterase inhibitors and NMDA antagonists only [9]. New A*β*-targeted immunotherapies have been tested in several clinical trials but without a clear clinical benefit [10]; therefore, no course-modifying treatment has been developed to date because of a lack of understanding of the fundamental mechanisms underlying AD, as well as the physiological role of amyloid peptides.

Senile plaques in AD patients and animal models consist of A*β*_1-40_ and A*β*_1-42_ (A*β*_1-42_ mainly in the core of early plaques and A*β*_1-40_ in vascular amyloid deposits) [11, 12]. It has been suggested that short A*β* fragments, such as A*β*_25-35_, constitute the biologically active forms of A*β* and are thus responsible for the neurotoxic properties of A*β*_1-40_ and A*β*_1-42_ [13]. A*β*_25-35_ may be expressed in AD brains [14–16] possibly from enzymatic cleavage of A*β*_1-40_ [15, 16]. Several studies indicate similar effects in the brain of either short or long forms of A*β* [13–15]. However, A*β*_25-35_ produces more acute toxic effects than A*β*_1-42_ because of its higher solubility [17], and it also has different effects on synaptic plasticity and intracellular pathophysiological mechanisms compared with A*β*_1-40_ and A*β*_1-42_ [18–20].

The hippocampus and entorhinal cortex are peculiarly susceptible to deleterious A*β* effects during early AD stages [21]. Hippocampal plasticity, necessary for learning and memory processes, is tuned by *θ* activity, which depends on acetylcholine release from the medial septum [2]. Moreover, the septum and hippocampus are reciprocally interconnected and functionally coupled through GABAergic and glutamatergic connections to form the septohippocampal system [2, 22]. Each of these neurotransmitters contributes to hippocampal rhythmicity [23]. Moreover, *θ* and *γ* activities are associated through inhibitory synapses between GABAergic parvalbumin interneurons and pyramidal neurons [24]. Such oscillatory activity, including phase-to-amplitude coupling of *θ* and *γ* activity, is necessary for adequately encoding and storing information in the cortex and hippocampus [25–27]. *In vivo* studies have shown the relevance of the CA3-CA1 synapse in associative learning and memory processing [28, 29] and its implications in AD through animal models [30]. In a very recent study, the electrophysiological activity of the CA3-CA1 region in humans was correlated with memory tasks (i.e., delayed match-to-sample) and it resembles synaptic hippocampal responses observed in rodents in the same areas [31]. Therefore, studies based on animal models may provide physiological information that could be applied in specific regions of clinical relevance in the human brain.

There are tight correlations among long-term potentiation (LTP) mechanisms, *θ*-*γ* oscillations, and hippocampal-dependent memories [32–34]. Event-locked oscillatory activity in hippocampal formation and hippocampus-related structures is necessary for learning and long-term memory processes, as well as for declarative and spatial memory functions, which are impaired in early stages of AD [3, 34, 35]. Accumulating evidence indicates that A*β* affects *θ*, *δ*, and *γ* bands in different preclinical models of AD [3, 36]. Similarly, EEG recordings in AD patients show pathological changes of network oscillations in a wide range of frequencies (i.e., *α*/*θ* ratio, *γ* coherence, *θ* and *δ* synchronization) [37, 38]. Studies using an animal model of AD (transgenic CRND8 mice) indicate that alterations in *θ*-*γ* cross-frequency coupling might be used as an early biomarker of AD [39]. In another study, acute LTP impairment by A*β*_1-42_ was related to alterations in oscillatory activity in *θ*-*γ* coupling at perforant path-dentate gyrus synapses [40]. Various studies have reported that changes in the spectral power of brain oscillations are related to LTP induction and expression [32, 33, 41, 42] and have shown a relationship between changes in single-synapse and network oscillation activity. Therefore, A*β*-induced LTP impairment might be associated with oscillatory activity changes in brain structures affected during initial stages of AD.

Several aggregated A*β* forms and configurations may explain variable effects during AD progression. Considering the huge differences between experimental models (*in vivo vs. in vitro*), time of exposure to A*β* (acute *vs.* chronic), and differences in A*β* aggregation states (monomeric, oligomeric, and fibrillary), the reported A*β* effects on neuronal activity have been divergent in terms of excitability, active and passive membrane properties, network activity, and neural plasticity [22, 43–47]. Despite the large number of studies, little is known about the effects of diverse soluble A*β* forms on oscillatory activity, excitability, or synaptic plasticity [47, 48]. The aim of the present study was, therefore, to evaluate A*β*_25-35_ and A*β*_1-40_ effects on hippocampal oscillations (power spectral density and phase-amplitude coupling) and basal transmission, variability, and long-term plasticity in cCA3-to-CA1 synapses. We found that LTP impairment induced by acute administration of soluble A*β* (A*β*_1-40_ and A*β*_25-35_) is associated with abnormal synaptic variability and increased power of *γ*-band and ripple network oscillations and derailed *θ*-to-*γ* phase-to-amplitude coupling in CA1. Such A*β*-induced disruption in synaptic plasticity and network activity may underlie abnormalities in information processing and memory encoding.

## 2. Material and Methods

### 2.1. Ethical Statement

All procedures performed on living animals were performed in conformance with Animal Research: Reporting *In Vivo* Experiments (ARRIVE) guidelines [49], following the Guide for the Care and Use of Laboratory Animals (8th edition, National Institutes of Health) and fulfilling the Colombian regulation (Law 84/1989 and Resolution 8430/1993). In addition, every experimental design and all procedures were approved by the Universidad del Rosario Ethics Committee.

### 2.2. Animals

Seventeen 16-20-week-old male Wistar rats, weighing 300 ± 30 g, were used as experimental subjects. Experimental animals were supplied by the Universidad Nacional de Colombia animal facilities. Animals were housed in a sound-attenuated room in polycarbonate cages, in groups of four, under controlled environmental conditions: 20 ± 1°C temperature, 50 ± 10% relative humidity, and 12 h light/dark cycle (lights on from 07:00 to 19:00). Animals had food and water available *ad libitum*. Experiments were performed in the morning. Special care was taken to minimize animal suffering and to reduce the number of animals used.

Sample size was calculated according to the following formula [50]: sample size = 2SD^2^(*Z*^∝/2^ + *Z*^*β*^)^2^/*d*^2^, where  SD = 6.85, *Z*^∝/2^ = 1.96 with ∝ = 0.05, *Z*^*β*^ = 0.84 with *β* = 0.2. Statistical power = 80%, and *d* = 14. The minimum sample number per group was 5.

### 2.3. Experimental Design and Timeline

Animals were randomly assigned to any of three groups: (1) control group (*n* =6), receiving vehicle microinjection; (2) A*β*_25-35_ group (*n* = 6), receiving A*β*_25-35_ peptide microinjection; and (3) A*β*_1-40_ group (*n* = 5), receiving A*β*_1-40_ peptide microinjection.

**Figure 1:**
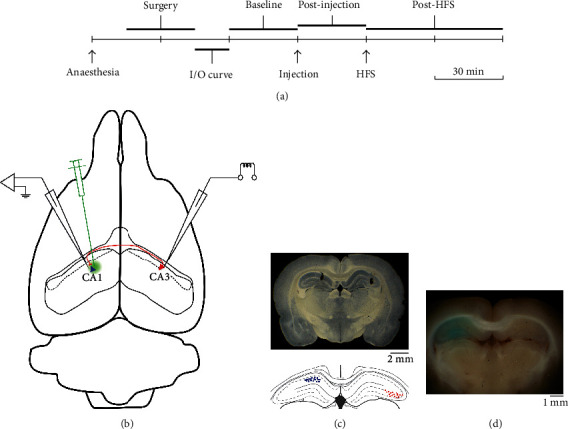
Experimental timeline and preparation. (a) Timeline indicating the experimental procedures during a recording session. (b) Diagram of a rat brain illustrating microelectrode cannula (for local field potential recording and microinjection in left CA1) and stimulating electrode (in right CA3) locations. (c) Panoramic photograph of an obliquely illuminated coronal brain slice containing representative electrolytic lesions made by passing current through recording (left) and stimulating (right) electrodes (upper panel), paired to a corresponding coronal diagram of the hippocampus (extracted and modified from the bregma: -3.6 mm diagram of Paxinos' rat brain atlas), summarizing recording (left, blue circles) and stimulating (right, red triangles) electrode placement in each experimental subject. (d) Panoramic photograph of a brain block containing methylene blue-coloured left hippocampus, attesting adequate diffusion of the injected solution.

In brief, each experiment proceeded as follows: (1) under general anaesthesia, recording and stimulation electrodes were stereotactically inserted in CA1 and contralateral CA3; (2) once stable responses were obtained, the input/output relationship was established; (3) 30 min baseline recording was done to characterize basal synaptic efficiency; (4) the designed solution was microinjected in CA1; (5) the microinjected solution's effect on synaptic transmission was characterized by recording synaptic responses for 30 min after microinjection; (6) high-frequency tetanic stimulation was done to induce long-term plasticity; and (7) the effect of high-frequency stimulation (HFS) on synaptic efficiency was characterized by recording synaptic responses for 60 min after HFS (Figure 1(a)).

### 2.4. Surgery

Under general anaesthesia induced with 1.5 g/kg of 25% urethane (Sigma-Aldrich, St. Louis, USA) and 10 mg/kg of xylazine (Rompun®, Bayer, Leverkusen, Germany) (intraperitoneal injection), the subject was placed in a rat stereotaxic frame (SR-6R, Narishige Inc., Tokyo, Japan). Nociceptive responses (tail prick and paw withdrawal reflex) were evaluated during the experiment. In case of slight motor response, an additional injection of urethane at 50% of the initial dose was administered. The respiratory and cardiac rates were monitored during the whole experiment, and a heating blanket was used to avoid hypothermia. Because we were interested in evaluating A*β* effects on NMDA-dependent synaptic plasticity, we chose urethane as the anaesthetic agent, taking into account that it has been reported to have no significant effects on NMDA-type glutamate receptors when given in intermediate doses [51, 52]; however, anaesthetic doses of urethane depress or even abolish 7-12 Hz atropine-resistant theta activity [53], but left 4-7 Hz atropine-sensitive theta oscillations unaltered. Longitudinal fronto-occipital incision, followed by connective and muscle tissue dissection, was used to expose the skull. Two holes were drilled in parietal bones: one for inserting a recording electrode aimed at left CA1 (stereotaxic coordinates from the bregma: AP = −3.8 mm, L = 2.5 mm, left) and one for inserting a stimulating electrode aimed at right CA3 (stereotaxic coordinates from the bregma: AP = −3.8 mm, L = 3.7 mm, right) [54]. In order to reduce variability between subjects, we always use the same electrode configuration. The dura mater was cut and removed through the holes in order to allow electrode insertion (Figure 1(b)).

### 2.5. *In Vivo* Extracellular Electrophysiology

A microelectrode cannula (a 5 M*Ω* impedance enamel-coated wire attached to a 25-G needle), for local field potential (LFP) recording and microinjection, was inserted 2.5 mm from the pial surface, through the skull hole for left CA1 (vide supra for stereotaxic coordinates), using a hydraulic micromanipulator (SM-25C, Narishige Inc., Tokyo, Japan). A stimulating concentric bipolar electrode was lowered 3.5 mm from the pial surface, through the skull hole for right CA3 (vide supra for stereotaxic coordinates), using a micromanipulator (SM-25A, Narishige Inc., Tokyo, Japan). A silver electrode was placed in neck musculature as a reference.

CA1 field activity was magnified (100x) using an AC-coupled preamplifier (NEX-1, Biomedical Engineering, New York, USA). The preamplified signal was then band-pass filtered (0.1 Hz and 10 kHz cut-off frequencies) and further amplified at 20x (yielding 2000x total gain). This conditioned signal was digitized using an analogue-to-digital converter (DigiData 1200, Molecular Devices, San José, USA) with 10 kHz sampling frequency and stored for offline analysis using commercial (Clampfit, Molecular Devices, San José, USA) and purpose-designed software.

CA3 was stimulated by applying 100 *μ*s monophasic square pulses, delivered at 0.33 Hz frequency, using a stimulus isolation unit (Isolator-11, Molecular Devices, San José, USA), controlled by a pulse generator (9514 Plus, Quantum Composers, Bozeman, USA). Stimulus intensity (100-400 *μ*A) was adjusted so as to obtain stable and reliable CA1 field excitatory postsynaptic potentials (fEPSP). The depth of the stimulating and recording electrodes from the pial surface was finely adjusted so as to obtain short latency fEPSP, with a waveform characteristic of CA1 stratum pyramidale.

Once a stable CA1 fEPSP was achieved, threshold stimulus intensity was established by decreasing the intensity to one-half the previous one until the fEPSP failure rate was equal to or higher than 50% (characteristically 50 to 200 *μ*A). Stimulus intensity was then successively doubled until fEPSP response saturation was attained (characteristically 4 to 8 times the threshold intensity). Then, the stimulus intensity required to obtain ~50% of maximal response (*I*_50_) was selected to evaluate short- and long-term plasticity from then on. Basal synaptic activity was characterized by recording CA3 stimulation-evoked fEPSP in CA1 (*I*_50_ intensity, 100 *μ*s duration, and 0.1 Hz frequency) for 30 min. Intrahippocampal microinjection effect on basal synaptic activity was characterized by recording such fEPSP for 30 min after microinjection. Long-term potentiation at cCA3-to-CA1 synapses was induced by delivering six trains (1 s length, 100 Hz frequency) at 60 s intertrain intervals (HFS). HFS effect on cCA3-to-CA1 synaptic efficiency was studied by recording left CA1 response to right CA3 stimulation (*I*_50_ intensity, 100 *μ*s duration, and 0.1 Hz frequency) for 60 min. Deep anaesthetic level was maintained throughout the whole recording session using supplementary anaesthetic doses (about every 4 hours) to attain stable and reliable activity in CA1 [55].

Once the recording session ended, a terminal dose of anaesthesia (urethane 2 g/kg and xylazine 10 mg/kg, intraperitoneal) was given and the location of stimulating and recording electrode tips was marked by passing continuous current through them delivered by a precision current source (Midgard, Stoelting, Wood Dale, USA). The subject's brain was removed and submerged in 4% paraformaldehyde for 3 days; then, 100 *μ*m thick coronal slices encompassing stimulating and recording sites were obtained using a vibratome (1000 Plus, Vibratome, Bannockburn, USA). The electrodes' position and diffusion trace of microinjection coloured solution were documented by examining the slices with a stereoscope (SZX16, Olympus, Tokyo, Japan) and taking digital photographs (Cybershot DSCW7, Sony, Tokyo, Japan) under oblique back-illumination (Figure 1(c)).

### 2.6. Preparation of Vehicle and A*β* Peptide Solutions

Among many soluble A*β* species, A*β*_25-35_ and A*β*_1-40_ (Sigma-Aldrich, St. Louis, USA) were chosen for the present work on the basis of their aggregation kinetics, neurotoxicity, and pathogenicity. On the one hand, postmortem examination of AD patients' brains yielded that A*β*_1-40_ accounts for approximately 90% of total A*β* peptide in senile plaques [56]. On the other hand, A*β*_25-35_ aggregates more rapidly and displays more neurotoxicity than A*β*_1-40_ [13, 57]. A*β*_25–35_ and A*β*_1-40_ peptides were prepared as previously described [22, 43, 45]. Briefly, peptides were dissolved in 0.9% normal saline solution with 0.5% methylene blue to 1.5 mM concentration and stored at -20°C. Aliquots were defrosted and incubated at 37°C for 24 h before experiments [58, 59]. The vehicle solution was therefore 0.9% normal saline with 0.5% methylene blue. Methylene blue was used to attest adequate diffusion in the hippocampal CA1 region of either vehicle or A*β* peptides (Figure 1(d)).

### 2.7. Intrahippocampal Microinjection

Once a baseline recording was obtained, a Hamilton syringe connected through 12-G tubing was used to inject 2 *μ*L of the designed solution (either vehicle, A*β*_25-35_, or A*β*_1-40_) at a 1 *μ*L/min rate through the microelectrode cannula inserted in the left hippocampal CA1 region. A*β*_25-35_ and A*β*_1-40_ dose (3 nM) and total injection volume (2 *μ*L) were chosen according to previous reports [60–63]. Stimulation and recording were restarted three minutes after microinjection in order to allow diffusion and prevent leakage of injected solution.

### 2.8. Data Analysis

**Figure 2:**
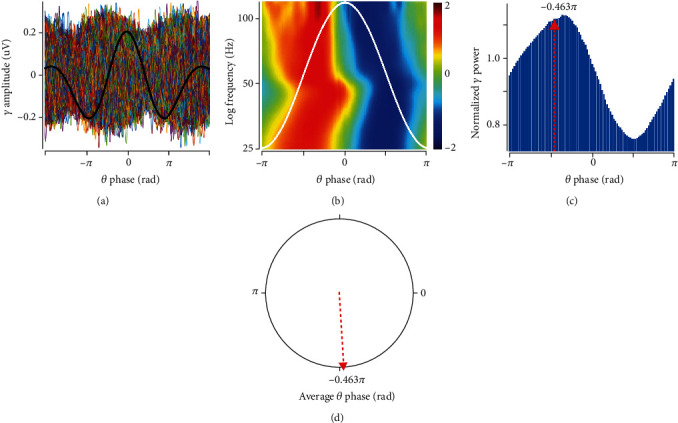
*θ*-to-*γ* phase-amplitude coupling (PAC) analysis process. (a) Filtered LFP in *θ* (black trace) and *γ* bands (coloured traces) are represented; *θ* trace is the average of *n* theta cycles, aligned around their maximum amplitude (downscaled to fit *γ* amplitude); each *θ* cycle-concurrent *γ* activity is presented in superposition using the abovementioned alignment. (b) Average normalized *γ* band scalogram (colour scale) relative to the *θ* phase (illustrated as a white trace). (c) Normalized *γ* band spectral power average as a function of the *θ* phase. (d) Average vector of (c) indicating dominant-phase *θ*-to-*γ* PAC.

Electrophysiological data analyses were planned to characterize the effects of both microinjected solutions on basal cCA3-to-CA1 synaptic responses, cCA3-to-CA1 long-term plasticity, and CA1 oscillatory activity. To do so, recordings were divided into 5 min windows around stimulation events and analysed in time, frequency, and time-frequency domains. Time domain analysis was related to fEPSP first component slope measurement. Frequency domain analysis was focused on calculating relative power spectral density (rPSD) in *δ* (0.5-3.9 Hz), *θ* (4-7.9 Hz), *α* (8-11.9 Hz), *β* (12-24.9 Hz), *γ* (25-120 Hz), HFO_1_ (121-250 Hz), and HFO_2_ (250-500 Hz) bands, using Welch's method. Time-frequency domain analysis was done by building scalograms for each window, using the Morse wavelet decomposition [64, 65]; then, *γ* band scalogram averages were triggered by each *θ* cycle time window to determine phase-amplitude coupling (PAC) [66] (Figure 2). Detailed information about mathematical data processing, which was done using self-written scripts using MATLAB R2017a® (The MathWorks, Inc., Natick, Massachusetts, USA), can be found in Supplementary Materials (available here).

### 2.9. Statistics

According to data distribution normality, determined using the Shapiro-Wilk test, long-term plasticity and PSD data from experimental groups were compared using either one-way ANOVA or Kruskal-Wallis one-way analysis of variance by rank modules of SigmaPlot 12.0 (Systat Software, Inc., San José, California, USA). Variability of cCA3-to-CA1 synaptic responses from experimental groups was compared using Levene's test [67]. The resulting angle from the average PAC vector in the experimental groups was compared using the MATLAB toolbox for circular statistics [68].

## 3. Results

### 3.1. A*β* Did Not Alter Synaptic Efficiency but Induced Changes in Synaptic Variability

**Figure 3:**
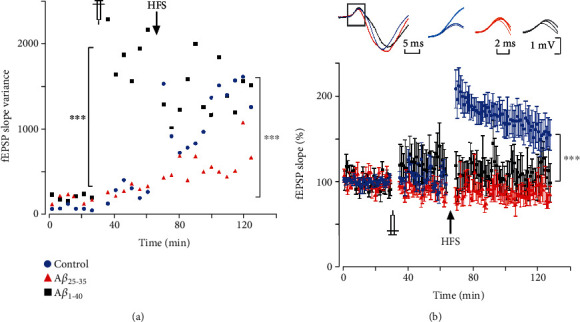
Intrahippocampal A*β* injections altered synaptic variability and impaired LTP in cCA3-to-CA1 synapse. Temporal evolution of slope variability (a) and magnitude (b) of cCA3 stimulation-evoked EPSP in CA1 recorded along three consecutive experimental stages, from left to right: (1) 30 min before peptide injection (baseline), (2) 30 min after intrahippocampal injection, and (3) after HFS (six 1 s, 100 Hz trains, delivered every 60 s). EPSP variability increased after intrahippocampal injection, being significantly higher in the A*β*_1-40_ group. HFS induced significant LTP in vehicle-injected subjects but not in A*β*-injected ones. In (a), each dot represents 5 min variance of slope; in (b), each dot illustrates 2 min mean ± standard error of the mean (SEM). Inset in (b): left—representative whole CA1 fEPSP average from each experimental group evoked by *I*_50_ stimuli delivered in contralateral CA3 (the region representing the monosynaptic component is outlined by a gray rectangular box); right—three sets of average traces (10 trials per average) of the monosynaptic component of cCA3 stimulation-evoked CA1 field potential obtained during baseline (thin line, dark colour), after intrahippocampal injection (intermediate line, intermediate colour), and after HFS (thick line, light colour) for each experimental group (control, blue; A*β*_25-35_, red; and A*β*_1-40_, black). ^∗∗∗^Significant differences between groups (*p* < 0.001). Data from each experimental group were normalized respecting the average value obtained during the last 15 min of baseline.

No significant difference was found between the experimental groups during baseline recording before (*H*_(2)_ = 0.153, *p* = 0.797, *n* = 16) or after intrahippocampal injection (*F*_(2, 13)_ = 1.541, *p* = 0.251, *n* = 16: Figure 3(a)). However, fEPSP slope variability significantly changed after intrahippocampal injection (*F*_(2,461)_ = 64.898, *p* < 0.001, *n* = 16); *post hoc* analysis showed that A*β*_1-40_ microinjection significantly increased cCA3-to-CA1 synaptic variability more than the other treatments (control *vs.* A*β*_25-35_: *F*_(1,317)_ = 0.004, *p* = 0.951, *n* = 11; control *vs.* A*β*_1-40_: *F*_(1,287)_ = 71.932, *p* < 0.001, *n* = 10; and A*β*_1-40_*vs.* A*β*_25-35_: *F*_(1,318)_ = 86.351, *p* < 0.001, *n* = 11). Variability progressively increased in control and A*β*_25-35_ groups after both injection (control: *F*_(1,606)_ = 19.232, *p* < 0.001, *n* = 5; A*β*_25-35_: *F*_(1,636)_ = 23.761, *p* < 0.0001, *n* = 6) and HFS (control: *F*_(1,438)_ = 63.522, *p* < 0.0001, *n* = 5; A*β*_25-35_: *F*_(1,438)_ = 49.588, *p* < 0.0001, *n* = 6). By contrast, even though variability in the A*β*_1-40_ group increased substantially after being injected (*F*_(1,606)_ = 299.08, *p* < 0.0001, *n* = 5), it did not change after HFS (*F*_(1,438)_ = 0.63, *p* = 0.428, *n* = 5).

### 3.2. A*β* Impaired Long-Term Synaptic Plasticity

HFS induced significant fEPSP slope increase in vehicle-injected subjects (from 100 ± 0.91% to 198.8 ± 14%, *H*_(2)_ = 9.5, *p* = 0.009, *n* = 5). By contrast, both A*β*_25-35_ and A*β*_1-40_ impaired such HFS-induced fEPSP slope increase (A*β*_25-35_: from 100 ± 1.29% to 89.45 ± 16%, *F*_(2, 17)_ = 0.504, *p* = 0.614, *n* = 6; A*β*_1-40_: from 100 ± 1.84% to 113.5 ± 25%, *F*_(2, 17)_ = 0.938, *p* = 0.418, *n* = 5; Figure 3(b)). Indeed, fEPSP slope change after HFS was significantly different between groups (*F*_(2, 15)_ = 17.741, *p* < 0.001, *n* = 16); *post hoc* analysis (Tukey's test) showed that vehicle-injected subjects displayed fEPSP slope increase significantly greater than A*β*_25-35_- and A*β*_1-40_-injected ones (control *vs.* A*β*_25-35_: *Q* = 8.146, *p* < 0.001, *n* = 11; control *vs.* A*β*_1-40_: *Q* = 6.083, *p* = 0.002, *n* = 10), while these later groups were not significantly different to each other (*Q* = 1.792, *p* = 0.437, *n* = 11). These results show that soluble A*β* microinjection impairs cCA3-to-CA1 long-term synaptic plasticity.

### 3.3. A*β*_25-35_ Induced Increase in *γ* and HFO_1_ Band Relative PSD

**Figure 4:**
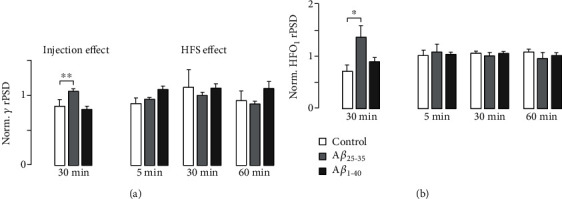
Peptide injection but not HFS increased rPSD in *γ* and HFO1 bands. Bar diagrams illustrating rPSD in *γ* (a) and HFO1 (b) bands. The left panel shows the effect of peptide or vehicle, 30 min after injection, normalized respecting baseline rPSD in the same bands. The right panel shows the effect of HFS normalized regarding rPSD calculated after injection in the same bands. Bars and whiskers represent each group's mean + SEM. ^∗^Significant difference respecting the control group during baseline (*p* < 0.05); ^∗∗^significant difference respecting each group after peptide injection (*p* < 0.01).

Intrahippocampal injection of A*β*_25-35_ induced significant increases in relative PSD (Figure 4) in *γ* (Figure 4(a), left column, *F*_(2, 11)_ = 8.237, *p* = 0.007, *n* = 17) and HFO_1_ (Figure 4(b), left column, *H*_(2)_ = 6.408, *p* = 0.029, *n* = 17) bands, but not in other bands. Neither A*β*_1-40_ nor vehicle injection induced significant changes in relative PSD in any band (Suppl. Table 1). In A*β*_25-35_-injected subjects, HFS did not induce additional changes in *γ* (Figure 4(a), right column; 5 min: *F*_(2, 11)_ = 3.036, *p* = 0.089; 30 min: *H*_(2)_ = 1.96, *p* = 0.403; and 60 min: *F*_(2, 11)_ = 1.502, *p* = 0.265), HFO_1_ (Figure 4(b), right column; 5 min: *F*_(2, 11)_ = 0.063, *p* = 0.940; 30 min: *F*_(2, 11)_ = 0.224, *p* = 0.803; and 60 min: *F*_(2, 11)_ = 0.341, *p* = 0.718), or any other band. HFS did not induce significant changes in relative PSD in any band in vehicle- or A*β*_1-40_-injected subjects (Suppl. Table 2). In summary, only A*β*_25-35_, which is the more toxic soluble species of A*β*, induced increased energy contribution in gamma and HFO_1_ bands, but HFS did not further modify such changes.

### 3.4. A*β* Injection Shifted *θ*-*γ* Phase-Amplitude Coupling but Impaired HFS-Induced Shift

To determine if the *γ* amplitude was linked to the *θ* phase at the hippocampus, different approaches can be used to calculate PAC. In this case, we calculated the average high-frequency *γ* power over the modulating low band in *θ* individual cycles. This method is especially useful when the modulating band is not constant over the length of the experiment. The power distribution was obtained by averaging the entire band, and this average gives a single value for the modulation between the pairs of frequency bands (*γ*-*θ*) (for details, see Figure 2 and Supplementary Materials).

**Figure 5:**
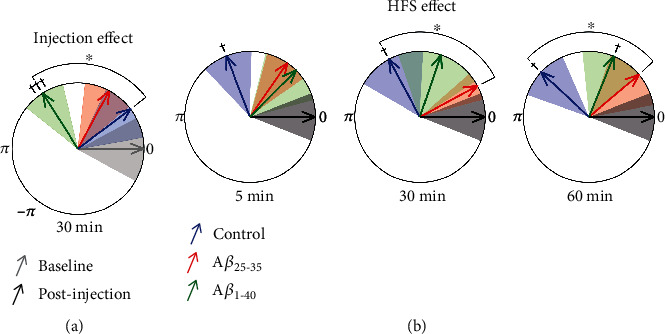
Peptide injection and HFS shifted the *θ*-to-*γ* PAC average phase vector. (a) Circular diagram illustrating the intrahippocampal microinjection-induced shift of the *θ*-to-*γ* PAC average phase vector for each group respecting the baseline vector. (b) Circular diagrams illustrating the time evolution (at 5, 30, and 60 min) of HFS-induced shift of the *θ*-to-*γ* PAC average phase vector for each group respecting the microinjection vector. Coloured arrows indicate average vector angles (control, blue; A*β*25-35, red; and A*β*1-40, green); correspondingly coloured shaded areas illustrate the standard error of angles for each group. Significant differences relative to the reference vector (^†^*p* < 0.05, ^†††^*p* < 0.001); significant differences between groups (^∗^*p* < 0.05).

A*β*_1-40_ injection induced a significant *γ* amplitude-modulating *θ* phase shift (~122°; *F*_(1, 8)_ = 37.220, *p* < 0.001); neither vehicle (*F*_(1, 6)_ = 1.59, *p* = 0.254) nor A*β*_25-35_ (*F*_(1, 9)_ = 3.26, *p* = 0.104) injection induced significant shifts in such modulating phase (Figure 5(a)). Planned intergroup comparisons showed that the A*β*_1-40_ injection-induced phase shift was significantly greater than vehicle injection-induced (*F*_(1, 6)_ = 7.71, *p* = 0.03) and A*β*_25-35_ injection-induced (*F*_(1, 9)_ = 4.76, *p* = 0.05) phase shifts; vehicle and A*β*_25-35_ injection-induced phase shifts were not significantly different to each other (*F*_(1, 7)_ = 0.43, *p* = 0.53).

Taking each group injection-induced phase shift as a reference, it was found that HFS induced a significant phase shift (~112-137°) in vehicle-injected subjects (Figure 5(b), blue arrows; *F*_(3, 16)_ = 4.47, *p* = 0.002); this phase shift persisted for up to 1 h after HFS (5 min: *F*_(1, 4)_ = 9.25, *p* = 0.038; 30 min: *F*_(1, 4)_ = 7.07, *p* = 0.05; and 60 min: *F*_(1, 4)_ = 13.11, *p* = 0.022). In A*β*_1-40_-injected subjects, HFS induced a smaller phase shift (~45-70°) that reached significance only 60 min later (*F*_(3, 16)_ = 1.21, *p* = 0.338; 5 min: *F*_(1, 8)_ = 0.72, *p* = 0.421; 30 min: *F*_(1, 8)_ = 0.82, *p* = 0.392; and 60 min: *F*_(1, 8)_ = 5.55, *p* = 0.046). By contrast, HFS did not induce a significant phase shift in A*β*_25-35_-injected animals (*F*_(3, 16)_ = 1.03, *p* = 0.4073; 5 min: *F*_(1, 8)_ = 2.75, *p* = 0.136; 30 min: *F*_(1, 8)_ = 0.92, *p* = 0.366; and 60 min: *F*_(1, 8)_ = 0.25, *p* = 0.631).

## 4. Discussion

This experiment's main findings were that, even though intrahippocampal microinjection of soluble species of A*β* did not change basal transmission, it significantly affected several other properties of cCA3-to-CA1 synapses: (1) A*β*_1-40_ enhanced basal synaptic variability significantly more than other treatments did but impaired HFS-induced variability increase; (2) A*β*_25-35_ injection significantly increased gamma and HFO1 band relative PSD; (3) both soluble amyloid beta peptides (A*β*_25-35_ and A*β*_1-40_) impaired HFS-induced LTP; and (4) A*β*_1-40_ injection induced a significant *γ* amplitude-modulating *θ* phase shift (~122°) but, as A*β*_25-35_ did, impaired the occurrence of a HFS-induced phase shift.

A*β* peptides have been repeatedly highlighted as crucial AD pathogenetic initiators. Although the underlying mechanism is not yet fully understood, some studies have indicated that A*β* can impair synaptic transmission and plasticity, leading to changes in spine density and, eventually, synaptic pruning [69–71]. We have found that intrahippocampal microinjection of soluble A*β* in anaesthetized rats affected cCA3-CA1 synapse variability and impaired long-term synaptic plasticity. Overall, such results concur with those of other studies, which have shown that high A*β* oligomer concentration interferes with synaptic efficiency and plasticity [61, 62, 72]. There is a mild increase in fEPSP slope variability after intrahippocampal injection of the vehicle. A similar effect has been observed in other types of *in vivo* preparations [73, 74], possibly due to a mechanical and osmotic effect of the saline solution. Another possibility of that change may involve methylene blue; however, the toxic effects of this molecule *in vitro* have been reported at concentrations higher than 100 *μ*M (around ten times our preparation) [75, 76]. Methylene blue might inhibit A*β* oligomerization in a dose-dependent manner (ranging from 0.01 to 445 *μ*M), but that effect is observed only after several days of incubation (4 to 8 days) [77]. We found that injection of A*β*_1-40_, but not A*β*_25-35_, induces significant increases in variability in cCA3-to-CA1 synaptic responses without significant changes in fEPSP slope. In agreement with our results, several *in vivo* studies found that the injection of different amyloid species did not affect hippocampal baseline synaptic potential amplitude or slope [60, 74, 78]. Synaptic variability is determined, among many factors, by presynaptic axonal noise, as well as release probability fluctuations [79]. On the one hand, A*β*_1-42_ has been found to induce spike widening, which would increase synaptic release due to increased calcium influx into presynaptic boutons [80]; A*β*_25-35_ has also been reported to produce spike broadening, but using doses one order of magnitude higher than the one used in our experiment [81]. On the other hand, A*β*_1-42_ oligomers depress release probability at CA3-CA1 synapses [82]; moreover, glutamatergic synaptic transmission could be either enhanced or reduced by A*β*_1-40_, depending on its concentration and the specific pyramidal cell type affected [44]. Such opposing mechanisms could explain the observed A*β*_1-40_-induced synaptic variability increase, without significant changes in average fEPSP slope. It is plausible that the A*β*_25-35_ concentration we used was not enough to induce significant changes in synaptic variability (however, it did affect other synaptic and network properties).

Although neither A*β*-intrahippocampal injection nor HFS induced significant CA1 global power spectrum changes (Suppl. Tables 3 and 4), specific frequency band rPSD computation evidenced that injection with A*β*_25-35_, but not A*β*_1-40_ nor HFS, induced a significant *γ* and HFO_1_ relative power increase. Transient (about 100 s) increases in *γ* and *θ* spectral power have been recorded in freely behaving adult rats' hippocampus immediately after LTP induction [32]. In the present experiment, such early PSD changes in control subjects were not detected given the 5 min window in our PSD analysis algorithm, which was not designed to detect short-lived changes.

Both *γ* oscillations and ripples (140-200 Hz) in the CA1 hippocampal region depend critically on the fast-spiking activity of parvalbumin- (PV-) expressing basket cells [83]. A*β* oligomers have been shown to directly interact with receptor tyrosine-protein kinase erbB-4, increasing its phosphorylation state [84]; erbB-4 activation increases PV interneuron-dependent oscillatory activity [85]. PV interneurons' increased activity, which has been described during early stages of AD models in association with subtle cognitive deficits [86, 87], seems to represent an initial adaptive response to A*β* oligomer deposition, which is followed later by PV interneuron dysfunction and more severe cognitive deficits [7, 88]. It is plausible that A*β*_25-35_ has more affinity than A*β*_1-40_ for erbB-4.

It has been found that *γ* band (25-120 Hz) activity increases in close association with locomotor behaviour [89, 90], working memory [91], and memory replay [92]. Hippocampal oscillations at frequencies higher than 100 Hz, also known as ripples (140-200 Hz), have been described to have several implications in cognitive processes [93]. In fact, ripples have been associated with learning and memory consolidation in humans and animals [94–97]. The observed soluble A*β* species-induced modifications in *γ* band and ripple oscillations in neural circuits are, therefore, associated with synaptic dysfunction and cognitive impairments [47, 98–100].

Both A*β* species used in our experiment (A*β*_25-35_ and A*β*_1-40_) impaired HFS-induced LTP in cCA3-to-CA1 synapses. This deleterious effect has been extensively reported, and many possible underlying mechanisms have been identified; among them, those pointing towards excitatory/inhibitory imbalance are relevant to our findings. A*β*-induced calcium dyshomeostasis underlies distorted synaptic transmission, plasticity, and oscillatory activity in the brain. However, depending on exposure time, brain region, oligomer type, and receptor subunits involved, soluble amyloid has been reported not only to inhibit calcium influx through NMDA receptors in cultured hippocampal [101] or cortical neurons [102] but also to increase NMDA-mediated calcium influx in mouse brains *in vivo* [103]. In addition, deleterious effects of A*β*_1-42_ and A*β*_1-40_ on NMDA function and LTP are reverted by a specific GLUN2B receptor antagonist [104]. Acute *in vivo* and *ex vivo* A*β*_1-42_ administration deteriorates GABA_B_-mediated inhibitory transmission in CA3-to-CA1 synapses and impairs HFS-induced LTP of excitatory [48] as well as inhibitory potentials [105], and such effects are reverted by pharmacological activation of G-protein-gated inwardly rectifying potassium (GirK) channels. A*β*_25-35_ has been found to act *ex vivo* as a GirK channel antagonist in CA3 pyramidal neurons [22]. A*β*-induced malfunction of NMDA and GirK channel conductance in pyramidal neurons might contribute, along with PV interneuron dysfunction, to hippocampal network instability, manifested through LTP impairment and aberrant rhythm generation, which may underlie subtle cognitive derailments observed during early AD stages.

Intrahippocampal injection of A*β*_1-40_ significantly shifted the resulting angle from the average *θ*-to-*γ* PAC vector. In vehicle-injected subjects, HFS induced a significant phase shift of the average *θ*-to-*γ* PAC vector persisting up to 60 min. Besides impairing HFS-induced LTP, both A*β*_25-35_ and A*β*_1-40_ blocked the phase shift of the average *θ*-to-*γ* PAC vector, with the A*β*_25-35_ effect persisting longer. Increases in *θ*-to-*γ* coupling have been described in freely behaving rats after HFS-induced LTP; in that experiment, acute A*β*_1-42_ treatment not only impaired LTP but also diminished *θ*-to-*γ* coupling [40]. *θ*-nested *γ* oscillations in CA1 depend on out-of-phase firing sequences of PV interneurons, pyramidal cells, somatostatin-positive (SST) neurons, and CA3-activated feedforward inhibitory interneurons during population *θ* oscillations, eventually opening windows for synaptic plasticity during specific *θ* phases [106]. Perisomatic inhibition by PV interneurons, associated with temporal silencing of feedforward inhibition acted by SST interneurons, allows calcium spike-associated plasticity; conversely, dendritic inhibition by feedforward interneurons prevents calcium spikes but facilitates pyramidal neuron output [106, 107]. Phase synchronization of firing in such a network depends on the relative contribution of PV (*γ* band) and SST (*θ* band) interneurons [108]; therefore, *θ*-to-*γ* coupling phase shift represents specific variations in such contributions. The observed A*β* injection-induced phase shift, as well as the impairment of HFS-induced phase shifts, may be due to its effect on PV interneurons [84, 85, 106] and on SST interneurons [108]. There may also be pyramidal cell contribution to such network dysfunction; in fact, A*β*_25-35_ reduces GABA_B_-dependent GirK channel activity in pyramidal neurons [22]; this may enhance excitatory pyramidal cell influence on PV and SST interneurons, which would further destabilize the network. Interestingly, in a recent clinical study, restoration of temporal cortex *θ*-to-*γ* PAC was associated with working memory performance improvement in older adults [109].

## 5. Conclusions

The present study results indicate that changes in the functional relationships between *θ*, *γ*, and ripple oscillatory network activity in the hippocampus are highly correlated with amyloid-induced synaptic plasticity dysfunction in a model of early amyloid-*β* pathology (similar to what has been reported in early AD stages). Taken together, these results show that intrahippocampal microinjection of soluble forms of A*β* affects synaptic variability and plasticity and modifies neural processing and network activity, changes that might underlie cognitive deficits observed in early AD models. A*β*-induced derailment of the tight functional relationship in hippocampal circuits between *θ* oscillation (controlled by SST interneurons as well as by medial septum and entorhinal cortex inputs) and *γ* activity (controlled by PV interneurons) implies a dysregulation of the crosstalk of cholinergic, glutamatergic, and GABAergic systems during early AD stages, leading to impaired information processing and encoding. Therefore, the abnormality in *θ*-to-*γ* PAC hereby described is worth evaluating as a putative early biomarker of A*β*-induced synaptic dysfunction in AD, long before neurodegeneration is established.

AD is a chronic and complex neurological disorder that involves several mechanisms (i.e., neuroinflammation and oxidative stress) additional to amyloid pathology. For that reason, it is important to start considering the role of *θ*-to-*γ* PAC in behavioural models of AD that involve tauopathy and selective chronic neurodegeneration, as well as to test it in further clinical trials through noninvasive electrophysiological methods in patients with mild cognitive impairment and major neurocognitive disorder.

## Data Availability

Additional data set can be sent by request. Detailed information of methods and results are included in Supplementary Materials.

## Supplementary Materials

Supplementary Materials This section includes supplementary methods and supplementary data (Supplementary Tables 1–4).
