# Supplementary material for: Acute Effects of Two Different Species of Amyloid-β on Oscillatory Activity and Synaptic Plasticity in the Commissural CA3-CA1 Circuit of the Hippocampus
Source: Neural Plast. 2020 Dec 18;2020:8869526. doi: 10.1155/2020/8869526 (PMC7765721; doi:10.1155/2020/8869526)
Supplement: Supplementary Materials — This section includes supplementary methods and supplementary data (Supplementary Tables 1–4). [file 8869526.f1.docx]

**Supplementary Material**

**Supplementary Methods**

*Time domain:*

CA3 stimulation-evoked fEPSP in CA1 slope was measured detecting the maximum of first time-derivative in the 5 ± 1 to 10 ± 1 ms interval after CA3 stimulation with a semi-automatic script developed *ad hoc*. Time evolution of fEPSP slope during the whole experiment was characterized by averaging every minute measurements (6/min) and expressing them as percentage of whole baseline mean (30 min).

*Frequency domain:*

Energy distribution by frequency was estimated using Welch’s method to determine power spectral density (PSD) (Welch, 1967). After DC removal, the signal was divided into 8 successive epochs ($x_{m})$with 50% overlap. Every signal epoch was defined by:

$$x_{m}\left( n \right)=w\left( n \right)x\left( n+rm \right), n=0,1,\ldots M-1, m=0,1,\ldots K)$$

Where: r, is the window hop size (size of the fast Fourier transform divided by overlap factor), K is the number of segments and M is the length of the segment. Each segment’s periodogram was given by:

$$P_{x_{m},M\left( \omega_{k} \right)}=\frac{1}{M}\left| {FFT}_{N,k(x_{m})} \right|^{2}$$

The periodogram of each epoch was tapered using a Hamming window and then averaged between epochs.

Welch’s estimate of power spectral density was calculated by:

$$\hat{S}_{x}^{W}\left( \omega_{k} \right)=\frac{1}{K}\sum_{m=0}^{K-1} P_{x_{m},M\left( \omega_{k} \right)}$$

To determine relative PSD of a given frequency band, the calculated PSD for such band was divided by the calculated PSD for all bands (from 0.5 to 500 Hz). Relative PSD was calculated in five periods: at baseline, 30 minutes after intrahippocampal injection, and 5, 30 and 60 minutes after HFS. To evidence injection and HFS effects for a given subject and to normalize it between subjects, post-injection value was expressed relative to baseline value, and post-HFS values were expressed relative to post-injection value before normalization.

*Time-Frequency domain:*

Since physiological oscillations vary as a function of time, wavelet decomposition was used to determine the time a given spectral change occurred (Le Van Quyen and Bragin, 2007). Wavelet is wave-like complexed-valued time-frequency scalable function $\psi(t)$ that acts as a family of localized filters and provided a powerful analysis of oscillatory signals. Analytic Morlet wavelets were used to determine time-frequency variability of the signal $x(t)$:

$$W_{\psi}(t,s)=\left\langle x\left( t \right),\psi_{\tau,s}(t) \right\rangle\frac{1}{\sqrt{s}}\int_{-\infty}^{\infty} x(t)\psi^{*}\left( \frac{t-\tau}{s} \right)dt$$

It is a series of normalized bandpass operations, where $\psi_{\tau,s}\left( t \right)=\frac{1}{\sqrt{s}}\psi\left( \frac{t-\tau}{s} \right)$ is scaled by S (Mallat, 2008). This operation was performed for the desired values to obtain a 3-dimensional representation of spectral power for every time-frequency point. The results were presented as colour-contour scalograms. Since power decreases nonlinearly as frequency increases, each frequency power was normalized relative to its maximum; therefore, relative power for each frequency at every time point was represented.

*Phase-Amplitude coupling (PAC):*

This method describes how a low frequency oscillation phase modulates a high frequency amplitude (Le Van Quyen and Bragin, 2007b). In the present work theta-to-gamma (θ-γ) PAC was analyzed. To determine if γ amplitude was linked to θ phase, raw signal was filtered using two Chebyshev type II band-pass filters isolating frequential components in θ and γ bands and eliminating DC level. Every θ cycle was detected, and a time-window was centered around cycle’s maximum (window size was standardized such as to contain a whole cycle). θ and γ components in such θ-centered time windows were averaged (**Fig. 2A**) to determinate if γ amplitude was affected by θ phase. In order to calculate the modulating phase (Cohen, 2008; Canolty and Knight, 2010), for each θ-centered time window θ oscillation phase (using Hilbert’s transform angle), and γ oscillation normalized scalogram were determined. θ phase was segmented in one hundred bins going from -π to π radians; for a given θ phase bin, the values of time-aligned normalized γ band scalogram in every window were taken and averaged for every frequency (25-120 Hz); then, a normalized γ band scalogram relative to θ phase was built using the resulting phase-frequency-power matrix (**Fig. 2B**). Then, for each θ phase bin, whole γ band power was obtained by adding the power of every frequency in γ band and plotted as histogram (Tort et al., 2010) (**Fig. 2C**). A representative vector was obtained by averaging whole γ band power between θ phase bins ($\varphi=\frac{1}{100}\sum_{n=1}^{100} A_{n}e^{i\emptyset_{n}}$), (**Fig. 2D**), such resultant single vector represents the phase of θ oscillations for which γ amplitude coupling is at its maximum (PAC angle). PAC angle was calculated at baseline, 30 minutes after the intrahippocampal injection, and 5, 30 and 60 minutes after the HFS protocol. PAC angle shift after injection was calculated respecting baseline; PAC angle shifts after HFS was calculated respecting post-injection value.

**Supplementary Data**

| **Frequency band** | Statistic (d.f.) | Value | p |
| --- | --- | --- | --- |
| **δ** | H_(2)_ | 2.539 | 0.311 |
| **θ** | F_(2,11)_ | 1.376 | 0.293 |
| **α** | F_(2,11)_ | 0.317 | 0.732 |
| **β** | H_(2)_ | 0.754 | 0.722 |
| **γ** | F_(2,11)_ | 8.237 | **0.007** |
| **HFO_1_** | H_(2)_ | 6.408 | **0.029** |
| **HFO_2_** | H_(2)_ | 3.893 | 0.144 |

**Supplementary Table 1. Injection effect on relative PSD:** Summary of ANOVA or ANOVA on ranks results, statistic (F or H) and p values, for inter-group comparison of relative PSD (normalized to baseline values) for each frequency band after intrahippocampal Aβ injection. Abbreviations: d.f., degrees of freedom. Significant results are stressed in bold characters.

| **Frequency band** |  | **5 min after HFS** | **30 min after HFS** | **60 min after HFS** |
| --- | --- | --- | --- | --- |
| **δ** | Statistic (d.f.) | H_(2)_ | F_(2,11)_ | H_(2)_ |
|  | Value | 1.404 | 1.39 | 1.31 |
|  | p | 0.545 | 0.289 | 0.569 |
| **θ** | Statistic (d.f.) | F_(2,11)_ | F_(2,11)_ | H_(2)_ |
|  | Value | 0.6 | 1.989 | 3.284 |
|  | p | 0.566 | 0.183 | 0.201 |
| **α** | Statistic (d.f.) | F_(2,11)_ | F_(2,11)_ | F_(2,11)_ |
|  | Value | 2.555 | 1.081 | 1.306 |
|  | p | 0.123 | 0.373 | 0.31 |
| **β** | Statistic (d.f.) | F_(2,11)_ | F_(2,11)_ | F_(2,11)_ |
|  | Value | 0.396 | 0.361 | 0.44 |
|  | p | 0.682 | 0.705 | 0.957 |
| **γ** | Statistic (d.f.) | F_(2,11)_ | H_(2)_ | F_(2,11)_ |
|  | Value | 3.036 | 1.96 | 1.502 |
|  | p | 0.089 | 0.403 | 0.265 |
| **HFO_1_** | Statistic (d.f.) | F_(2,11)_ | F_(2,11)_ | F_(2,11)_ |
|  | Value | 0.0625 | 0.224 | 0.341 |
|  | p | 0.94 | 0.803 | 0.718 |
| **HFO_2_** | Statistic (d.f.) | F_(2,11)_ | F_(2,11)_ | F_(2,11)_ |
|  | Value | 0.696 | 0.0769 | 0.679 |
|  | p | 0.519 | 0.927 | 0.527 |

**Supplementary Table 2. HFS effect on relative PSD:** Summary of ANOVA or ANOVA on ranks results, statistic (F or H) and p values, for inter-group comparison of relative PSD (normalized to post injection recordings) for each frequency band after HFS protocol. Abbreviations: d.f., degrees of freedom.

| **Frequency band** | **Statistic (d.f.)** | **Value** | **p** |
| --- | --- | --- | --- |
| **δ** | F_(2,11)_ | 0.62 | 0.557 |
| **θ** | F_(2,11)_ | 2.02 | 0.179 |
| **α** | F_(2,11)_ | 1.28 | 0.317 |
| **β** | F_(2,11)_ | 2.16 | 0.162 |
| **γ** | F_(2,11)_ | 1.58 | 0.25 |
| **HFO_1_** | F_(2,11)_ | 1.06 | 0.378 |
| **HFO_2_** | H_(2)_ | 1.274 | 0.562 |

**Supplementary Table 3. Injection effect on PSD:** Summary of ANOVA or ANOVA on ranks results, statistic (F or H) and p values, for inter-group comparison of PSD (normalized to baseline) for each frequency band after intrahippocampal injection of Aβ. Abbreviations: d.f., degrees of freedom.

| **Frequency band** |  | **5 min after HFS** | **30 min after HFS** | **60 min after HFS** |
| --- | --- | --- | --- | --- |
| **δ** | Statistic (d.f.) | H_(2)_ | H_(2)_ | H_(2)_ |
|  | Value | 0.811 | 1.547 | 2.833 |
|  | p | 0.697 | 0.499 | 0.265 |
| **θ** | Statistic (d.f.) | H_(2)_ | H_(2)_ | H_(2)_ |
|  | Value | 0.741 | 1.989 | 2.074 |
|  | p | 0.727 | 0.4 | 0.381 |
| **α** | Statistic (d.f.) | H_(2)_ | H_(2)_ | H_(2)_ |
|  | Value | 1.684 | 0.495 | 0.741 |
|  | p | 0.461 | 0.809 | 0.727 |
| **β** | Statistic (d.f.) | H_(2)_ | H_(2)_ | F_(2,11)_ |
|  | Value | 0.23 | 0.373 | 1.311 |
|  | p | 0.903 | 0.852 | 0.309 |
| **γ** | Statistic (d.f.) | H_(2)_ | F_(2,11)_ | F_(2,11)_ |
|  | Value | 4.84 | 0.534 | 1.736 |
|  | p | 0.085 | 0.601 | 0.221 |
| **HFO_1_** | Statistic (d.f.) | F_(2,11)_ | H_(2)_ | H_(2)_ |
|  | Value | 1.093 | 1.602 | 3.24 |
|  | p | 0.369 | 0.483 | 0.214 |
| **HFO_2_** | Statistic (d.f.) | F_(2,11)_ | F_(2,11)_ | H_(2)_ |
|  | Value | 1.747 | 0.209 | 2.888 |
|  | p | 0.219 | 0.815 | 0.251 |

**Supplementary Table 4. HFS effect on PSD:** Summary of ANOVA or ANOVA on ranks results, statistic (F or H) and p values, for inter-group comparison of relative PSD (normalized to post injection recordings) for each frequency band after HFS protocol. Abbreviations: d.f., degrees of freedom.

**Supplementary Material References**

Canolty RT, Knight RT (2010) The functional role of cross-frequency coupling. Trends Cogn Sci (Regul Ed) 14:506–515.

Cohen MX (2008) Assessing transient cross-frequency coupling in EEG data. J Neurosci Methods 168:494–499.

Le Van Quyen M, Bragin A (2007) Analysis of dynamic brain oscillations: methodological advances. Trends Neurosci 30:365–373.

Mallat S (2008) A Wavelet Tour of Signal Processing: The Sparse Way. Academic Press. Chapter 1.2.2 Wavelet Transform 25-28.

Tort ABL, Komorowski R, Eichenbaum H, Kopell N (2010) Measuring phase-amplitude coupling between neuronal oscillations of different frequencies. J Neurophysiol 104:1195–1210.

Welch P (1967) The use of fast Fourier transform for the estimation of power spectra: A method based on time averaging over short, modified periodograms. IEEE Transactions on Audio and Electroacoustics 15:70–73.
